# Interpretation of P53 Immunohistochemistry in Endometrial Carcinomas: Toward Increased Reproducibility

**DOI:** 10.1097/PGP.0000000000000488

**Published:** 2018-12-14

**Authors:** Martin Köbel, Brigitte M. Ronnett, Naveena Singh, Robert A. Soslow, C. Blake Gilks, W. Glenn McCluggage

**Affiliations:** Department of Pathology and Laboratory Medicine, University of Calgary, Calgary, Alberta (M.K.); Department of Pathology, Vancouver General Hospital, Vancouver, British Columbia (C.B.G.), Canada; Department of Pathology and Gynecology and Obstetrics, The Johns Hopkins Medical Institutions, Baltimore, Maryland (B.M.R.); Department of Pathology, Memorial Sloan Kettering Cancer Center, New York, New York (R.A.S.); Department of Cellular Pathology, Barts Health NHS Trust, London (N.S.); Department of Pathology, Belfast Health and Social Care Trust, Belfast (W.G.M.), UK

**Keywords:** Endometrial carcinoma, p53, *TP53*, Immunohistochemistry, Interpretation

## Abstract

P53 immunohistochemistry has evolved into an accurate surrogate reflecting the underlying *TP53* mutation status of a tumor, and has utility in the diagnostic workup of endometrial carcinomas. Recent work predominantly carried out in tubo-ovarian high-grade serous carcinoma has revealed 4 main patterns of p53 staining (normal/wild-type, complete absence, overexpression, and cytoplasmic); the latter 3 patterns are variably termed abnormal/aberrant/mutation-type and are strongly predictive of an underlying *TP53* mutation. The aim of this review is to provide practical advice to pathologists regarding various aspects of p53 immunohistochemical staining. These include laboratory methods to optimize staining, a description of the different patterns of staining, advice regarding the interpretation, and reporting of p53 staining and practical uses of p53 staining in endometrial carcinoma diagnosis. Illustrations are provided to aid in the interpretational problems.

The mutational status of *TP53* is the single most important molecular factor, which predicts prognosis in endometrial carcinomas, with the presence of a *TP53* mutation being associated with an unfavorable outcome 1,2. The *TP53* mutation status may be used clinically in different ways such as aiding in the distinction between serous and endometrioid histotype 3,4, predicting outcome within a given histotype 1,5 or predicting outcome across several histotypes 2. The value of p53 in these scenarios is discussed in several other papers in this issue. As most pathologists do not have access to *TP53* sequencing, they use p53 immunohistochemistry, which is quick, easy to perform, and inexpensive, as a surrogate for *TP53* mutational analysis. Hence, p53 immunohistochemistry is very commonly utilized on endometrial carcinoma samples.

The aim of this review is to provide practical advice to pathologists regarding various aspects of p53 immunohistochemical staining. These include laboratory methods to optimize staining, a description of the different patterns of staining, advice regarding the interpretation, and reporting of p53 staining and practical uses of p53 staining in endometrial carcinomas.

## DIFFERENT PATTERNS OF P53 STAINING

It has long been recognized that nonsynonymous *TP53* missense mutations result in nuclear accumulation of p53 protein that can be detected as overexpression by immunohistochemistry. This is in the form of diffuse strong nuclear positivity involving at least 80% of the tumor cells but usually almost 100%. With increased refinement of immunohistochemistry, we, and others, observed other abnormal p53 expression patterns that correlate with the presence of a *TP53* mutation. Although most of this work has been performed in tubo-ovarian high-grade serous carcinomas, these patterns are also found in other tumor types 6,7 and we believe that identical staining protocols and interpretational cut points apply for endometrial carcinomas. However, validation studies in endometrial carcinomas are necessary.

**FIG. 1:**
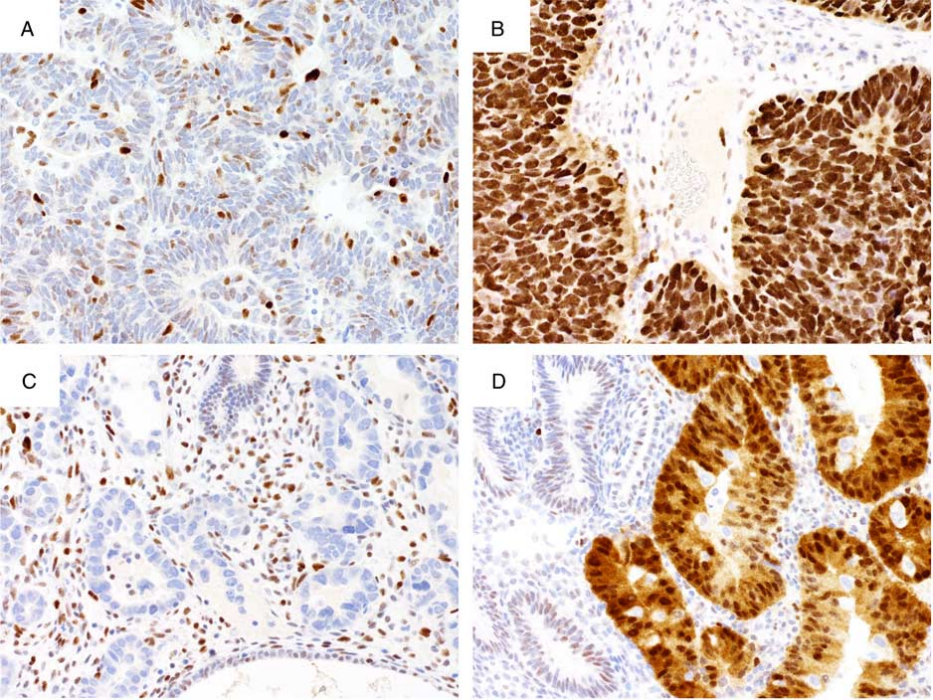
Different patterns of p53 expression. (A) Endometrial endometrioid carcinoma showing normal wild-type pattern of p53 expression with variable proportion of tumor cell nuclei staining with variable intensity. Note, this wild-type pattern should not be reported as “positive,” because this is ambiguous reporting language. (B) Endometrial endometrioid carcinoma, grade 3, with overexpression, showing strong staining in virtually all tumor cell nuclei, much stronger compared with the internal control of fibroblasts in the center. Note, there is some cytoplasmic background indicating that this staining is quite strong but this should not be interpreted as abnormal cytoplasmic pattern. (C) Endometrial serous carcinoma showing complete absence of p53 expression with internal control showing moderate to strong but variable staining. Note, wild-type pattern in normal atrophic glands at 12 and 6 o’clock. (D) Endometrial endometrioid carcinoma showing cytoplasmic p53 expression with internal control (stroma and normal endometrial glands) showing nuclear wild-type pattern. The cytoplasmic pattern is accompanied by nuclear staining of similar intensity.

A 3-tier system has been recommended for p53 immunohistochemistry interpretation with overexpression and complete absence (which requires the presence of a positive internal control with staining of non-neoplastic cells such as lymphocytes, fibroblasts, or endothelial cells) both interpreted as abnormal/aberrant/mutation-type, in contrast to the normal/wild-type pattern with p53 expression levels in between these extremes 6,7. Wild-type staining is characterized by an admixture of negative cells, weakly and strongly positive cells. Subsequent studies using ovarian carcinomas validated that optimized immunohistochemistry agrees extremely well (specificity up to 100%) with the underlying *TP53* mutation status 8–10. In other words, if the p53 staining pattern is abnormal (aberrant/mutation-type) there is almost certainly an underlying *TP53* mutation. Notably, some splice site mutations or truncating mutations (the latter characterized by c-terminal stopgain) can result in detectable (but nonfunctional) p53 protein yielding a normal wild-type staining pattern. This occurs in ∼5% of tubo-ovarian high-grade serous carcinomas. With optimized immunohistochemistry, a fourth uncommon p53 staining pattern was observed. This cytoplasmic pattern is characterized by an unequivocal cytoplasmic staining, which is accompanied by a variable nuclear staining. Strong diffuse nuclear overexpression with low-intensity cytoplasmic background should interpreted as overexpression and not cytoplasmic. In tubo-ovarian high-grade serous carcinomas, the cytoplasmic pattern is associated with mutations disrupting the nuclear localization domain of the p53 protein 9. Thus, there are currently 4 patterns of p53 staining (Fig. 1), which correlate with the *TP53* mutational status, resulting in 2 main corresponding interpretational categories (Table 1). Table 1 shows the percentages of the different staining patterns seen in tubo-ovarian high-grade serous carcinomas, where *TP53* mutations are ubiquitous.

**TABLE 1:**
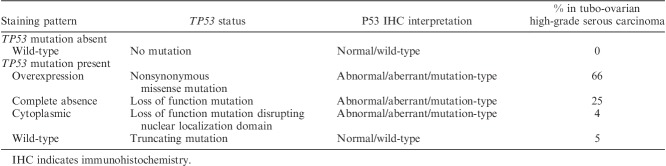
p53 immunohistochemical staining patterns observed in tubo-ovarian high-grade serous carcinoma

While immunohistochemistry can accurately predict the *TP53* mutation status and high interobserver agreement can be achieved with training 11, significant variation regarding the interpretation of p53 immunohistochemistry is still observed in practice. In the following sections, we review difficult areas in the interpretation of p53 immunohistochemistry, including interpretational difficulties between the normal wild-type staining versus the 3 abnormal staining patterns. We also discuss problems with heterogenous staining (defined as subclonally abnormal staining within wild type staining) that can sometimes be seen in endometrial carcinomas. We recommend that p53 staining should not be reported as positive or negative as this is confusing and ambiguous terminology; rather the pattern of staining should be reported as wild-type or abnormal/aberrant/mutation-type and the pattern of the latter described.

## INTERPRETATIONAL DIFFICULTIES: NORMAL WILD-TYPE VERSUS ABNORMAL OVEREXPRESSION

**FIG. 2:**
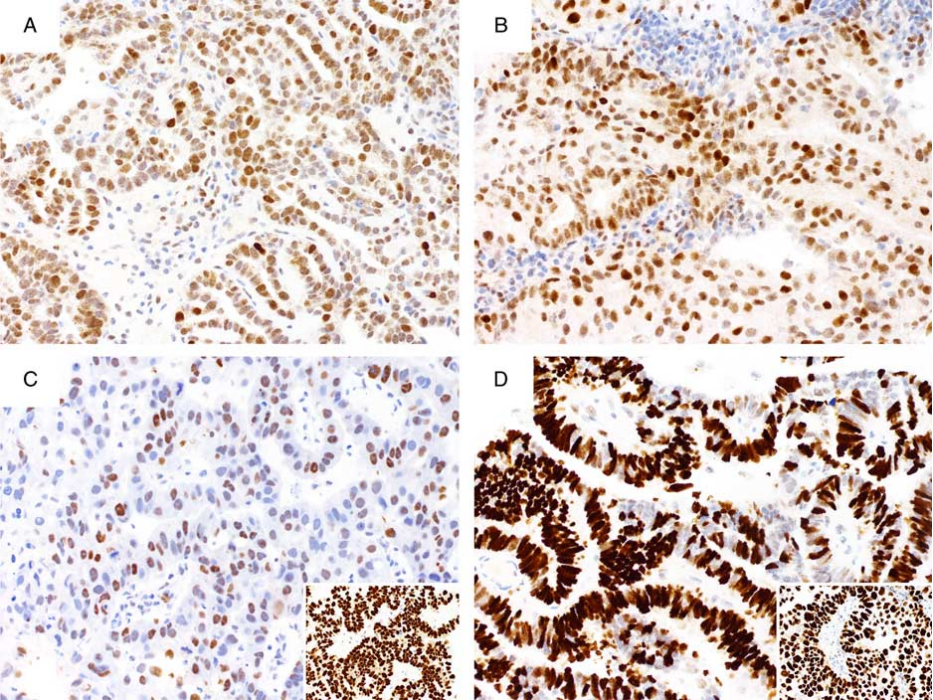
(A, B) Two low-grade endometrial endometrioid carcinomas with p53 staining in the majority of tumor cell nuclei but with variable intensity (some staining strong, some moderate, some weak, few negative). The intensity is within the range of the internal control but less intense compared with Figure 1B (same protocol). There is some cytoplasmic blush suggesting that the staining is bordering on too strong. (C, D) Two endometrial serous carcinomas with areas of “mosaic” staining bordering wild-type pattern (more in C than D) that show otherwise abnormal overexpression pattern in the remainder of the sample (see inset). The distinction of spurious “mosaic” staining due to poor fixation from true heterogenous staining (compare with Fig. 4) can be challenging.

Currently the main use of p53 immunohistochemistry is to predict the presence or absence of *TP53* mutation rather than a specific group of mutation, which may become of interest in the future when certain *TP53* mutations may become targetable 12. The normal wild-type pattern can show a significant range of staining from only very few tumor cell nuclei positive to the majority of nuclei being positive. The level of wild-type p53 expression is dependent on the cellular state of differentiation and related to the proliferative activity. Tumors with a higher proliferation index often show more p53 staining and tumors with so-called “high” wild-type staining may be confused with overexpression. Figures 2A and B show examples of tumors that were stained with a high p53 antibody concentration (see below). To guide interpretation, the p53 expression levels should be compared with the internal control (these cases show clear p53 staining in normal stromal fibroblasts, endothelial cells, and lymphocytes), and also to the expected overexpression pattern for this protocol (these cases, eg, stain weaker compared with Fig. 1B). In these tumors, the intensity of the nuclear staining is variable with a few nuclei exhibiting strong staining, most moderate to weak staining and some being negative. Taken together, the cases in Figures 2A and B are interpreted as wild-type, albeit quite “high” wild-type.

In contrast, some cases with nonsynonymous *TP53* mutations can show a lesser degree of p53 staining than expected for overexpression. Figures 2C and D show 2 endometrial serous carcinomas with areas of lower p53 staining compared with the remainder of the tumor shown in the inset. Preanalytical factors (such as delayed fixation resulting in antigen degradation, which is more common in hysterectomy than biopsy specimens) are the presumed cause for areas with lower expression in these cases. This should not be interpreted as heterogenous expression, as defined below, or wild-type staining. In general, stronger p53 staining is more resilient against fixation issues and usually provides the same staining when comparing endometrial biopsy with hysterectomy specimens. Conceivably, real molecular alterations could also explain these “mosaic” patterns, for example splice site mutation which may have an unpredictable effect on expression from 1 tumor cell to another, or low allelic frequency of *TP53* mutation in some tumor areas; however, such changes appear to be rare.

## INTERPRETATIONAL DIFFICULTIES: NORMAL WILD-TYPE VERSUS ABNORMAL COMPLETE ABSENCE AND NORMAL WILD-TYPE VERSUS ABNORMAL CYTOPLASMIC

**FIG. 3:**
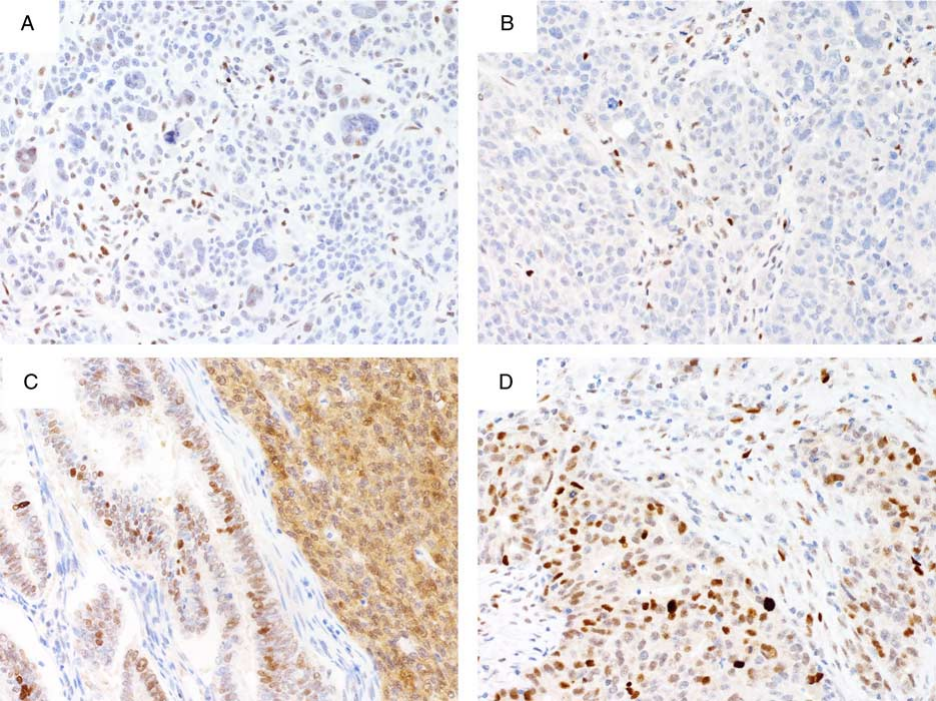
(A, B) Endometrial serous carcinoma with complete absence pattern of abnormal p53 expression stained on 2 different platforms. (A) Nonspecific nuclear staining interpreted as wild-type pattern; (B) shows complete absence of nuclear staining but a weak cytoplasmic blush indicating staining bordering on too strong. (C) Endometrial endometrioid carcinoma with wild-type staining with slight cytoplasmic blush on the left and true abnormal cytoplasmic staining on the right (compare with low-power view in Fig. 4C). The true abnormal cytoplasmic staining is accompanied by a variable nuclear staining of similar intensity but not strong diffuse. (D) Endometrial endometrioid carcinoma with wild-type pattern showing weak cytoplasmic staining probably due to too strong staining. This should not be interpreted as abnormal cytoplasmic staining.

The distinction of wild-type versus complete absence does not usually result in problems in interpretation as long as the tissue is well fixed and the assay sufficiently optimized to consistently stain normal cells. It is important to see adequate staining of internal controls (fibroblasts, endothelial cells, or lymphocytes) before making a diagnosis of abnormal complete absent p53 staining. Cases without a positive internal control are regarded as uninterpretable, and this may be more problematic on scant biopsies with minimal tissue represented. We also draw attention to an artifact, that is, encountered occasionally with the more sensitive p53 immunostaining methods; in some instances, a nonspecific nuclear blush can be present, which could be misinterpreted as wild-type in cases of true complete absence (Figs. 3A, B).

The cytoplasmic pattern of p53 staining has only recently been recognized as a distinct abnormal expression pattern (aberrant/mutation-type), which is occasionally seen with optimized immunohistochemistry, and experience with this pattern of staining is limited. The identification of this pattern of staining is very dependent on the immunohistochemical protocol because it is probably not seen with weaker staining (see below). We have encountered occasional cases with variable nuclear expression and a cytoplasmic blush where the distinction between wild-type and CY staining can be difficult (Figs. 3C, D). Truly abnormal CY staining should be definite, and not an equivocal blush, which can be ignored.

Interpretational difficulty may also arise within the various abnormal p53 staining patterns, although it seems impossible to confuse overexpression with complete absence. Of note, we have seen 1 tubo-ovarian high-grade serous carcinoma that showed overexpression on a pretreatment biopsy and a combination of complete absence and overexpression on the surgical specimen indicating either different clonal origin or tumor progression with acquisition of a second loss of function *TP53* mutation that resulted in a changed immunostaining pattern 13.

Although there are undoubtedly challenges with interpretation of p53 immunohistochemistry, equally there are also issues around quality assurance for genetic testing such that *TP53* mutation testing may not be totally reliable. For example, in external proficiency testing for *KRAS* mutations in colorectal carcinoma, which is much less technically challenging than *TP53* mutation analysis, >25% of laboratories had errors in 1 or more of 10 test samples 14.

## HETEROGENOUS P53 EXPRESSION

**FIG. 4:**
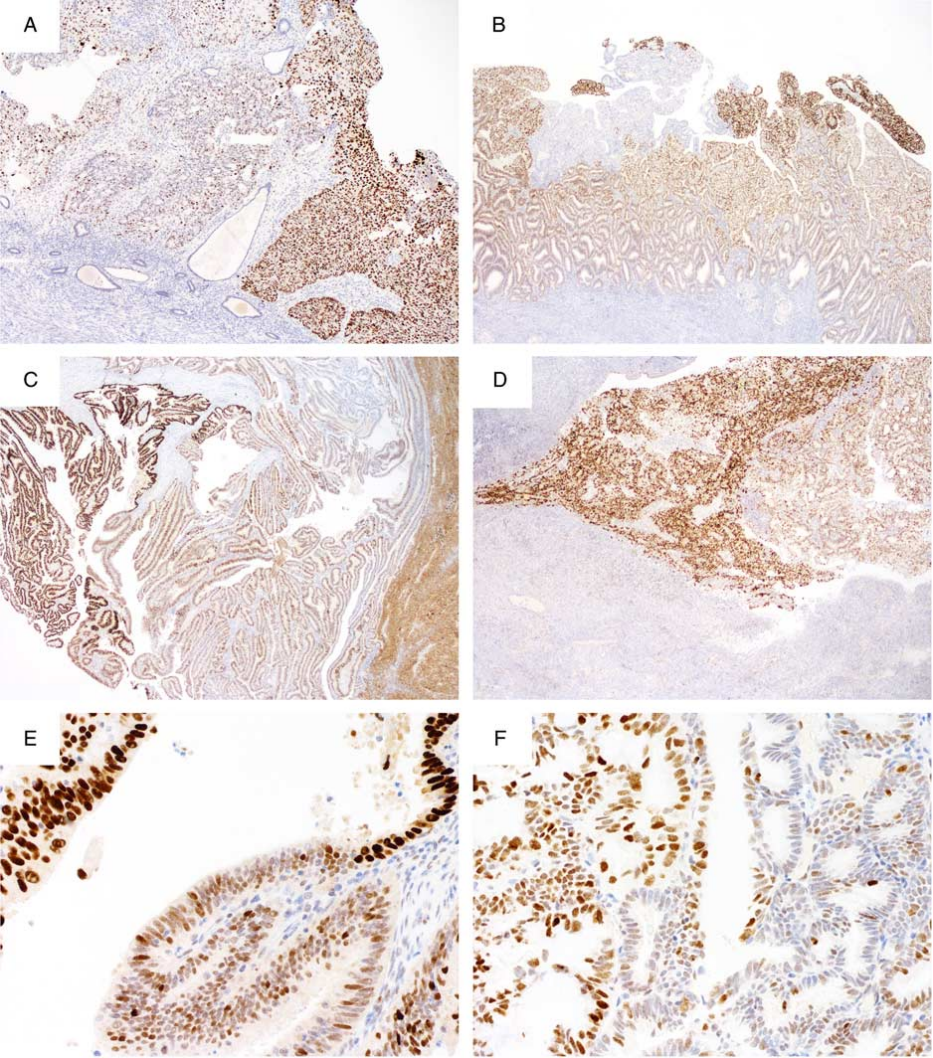
(A) Endometrial endometrioid carcinoma showing a combination of wild-type pattern on the left and overexpression on the right (normal+abnormal=heterogenous). (B) Endometrial endometrioid carcinoma with heterogenous staining (normal wild-type pattern+abnormal overexpression and complete absence). (C) Endometrial endometrioid carcinoma with heterogenous staining (normal wild-type pattern and abnormal overexpression and cytoplasmic staining). (D) Endometrial endometrioid carcinoma with heterogenous staining (normal wild-type pattern and abnormal overexpression and complete absence). (E) High power from (C) showing transition from overexpression to wild-type pattern. (F) Endometrial endometrioid carcinoma with variable wild-type pattern (not heterogenous), “high” wild-type on left versus “low” wild-type on the right.

In contrast to endometrial serous carcinomas, where *TP53* mutation is the early founder mutation and therefore present in any subclone, there are some endometrioid carcinomas with a mutator phenotype (either *POLE* ultramutated or mismatch repair deficient hypermutated) that can acquire a *TP53* mutation later in the course. Such a subclonal *TP53* mutation may result in heterogenous p53 expression characterized by areas of normal wild-type and areas with abnormal mutation-type staining patterns (Fig. 4). Traditionally, some of those tumors have been diagnosed as “mixed” endometrial carcinomas but they are probably better characterized as endometrioid carcinomas, particularly if endometrioid-like alterations (mismatch repair deficiency, loss of PTEN, or ARID1A) are detected 15. Interpretational difficulties in p53 staining can arise in determining whether these subclonal foci are indeed a distinct pattern (Fig. 4E) or whether this represents variability in the intensity of wild-type staining not reaching the threshold for abnormal overexpression (i.e. areas of “high” wild-type) (Fig. 4F) or variability in overexpression (Figs. 2C, D). As a potential pitfall, we have seen cases in which the abnormal component of heterogenous staining was present on 1 tumor section but not another. This raises the possibility that such a case may be erroneously classified as a serous carcinoma while it in fact it may be a *POLE* mutated endometrioid carcinoma. Hence, if morphologic features suggestive of *POLE* mutations are seen and p53 staining is abnormal, it may be useful to stain >1 tumor section 16,17.

## RECOMMENDATION FOR p53 IMMUNOHISTOCHEMISTRY OPTIMIZATION

The DO7 clone is the most widely used p53 antibody but there are several other high-quality p53 antibodies available. Each laboratory must establish an immunohistochemical protocol with high sensitivity and specificity using appropriate controls. For p53, these include both external and internal controls. As external controls, a “low expressor” positive control to assess the lower limit of detection along with a “high expressor” positive and a negative tissue control are recommended by immunohistochemistry proficiency testing programs (eg, NordiQC, UK NEQAS, CIQC) 18. The typical “high expressor” positive control is a high-grade serous carcinoma with overexpression. Tonsil or colon (serving both as a “low expressor” positive and a negative control) allow gauging of immunohistochemical protocols and should be considered for inclusion as on-slide controls. In the tonsil, there is variably intense nuclear staining of scattered keratinocytes in the lower third of the squamous epithelium and in the germinal center B cells (≥20%), whereas the upper squamous layers should be negative and only occasional cells of the mantle zones of secondary follicles (<10%) should stain. In the colon (as well as appendix), there is variably intense nuclear staining in scattered epithelial cells in the basal crypts but no staining of the luminal epithelial cells. An advantage of p53 immunohistochemistry is that an internal “low expressor” positive control is present in almost any tissue (fibroblasts, endothelial cells, or lymphocytes), with variably intense nuclear staining in scattered cells. This internal control is invaluable as it provides information not only about analytical but also preanalytical factors. As discussed previously, consistent staining of the internal control is a prerequisite for interpretation of the complete absence pattern.

**FIG. 5:**
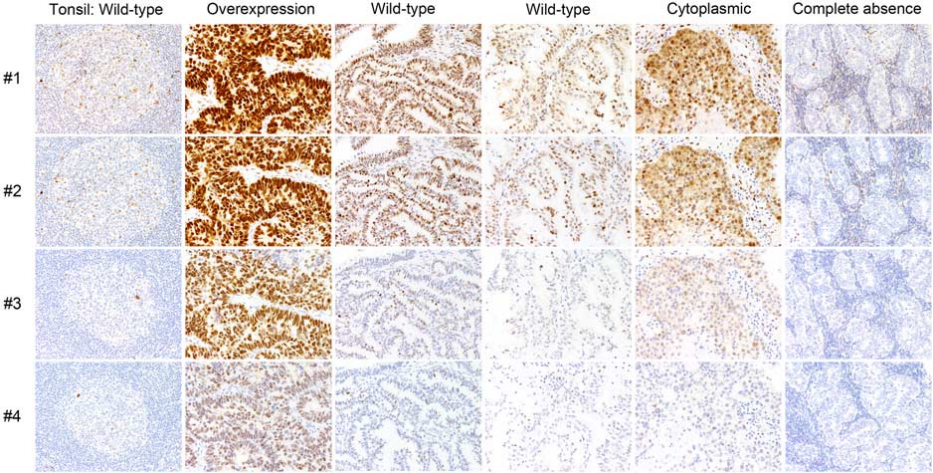
Staining results from 4 immunohistochemistry platforms: protocol #1 used EnVision Flex target retrieval solution (TRS), high pH, ready to use (RTU) primary antibody clone DO7 for 30 min with 10 min linker. Protocol #2 used the same as #1 except the RTU primary antibody clone DO7 was diluted (1/5). Protocol #3 used EnVision Flex TRS, high pH, RTU primary antibody clone DO7 for 20 min without linker (Dako vendor protocol). Protocol #4 used the same as #3 except the RTU primary antibody clone DO7 was diluted (1/10). Six specimens representing the 4 p53 staining patterns in columns from left to right: wild-type pattern in a germinal center of tonsil (“low expressor” positive control), overexpression in an endometrial serous carcinoma (“high expressor” positive control), 2 wild-type endometrial endometrioid carcinomas showing the range of wild-type expression, cytoplasmic and complete absence of staining (note presence and absence of internal control).

In Figure 5, we illustrate the influence of different immunohistochemical protocols on the staining results. Each column represents the same case stained with 4 different immunohistochemical protocols using the same DO7 antibody on the same Dako Omnis platform but varying primary antibody dilution and amplification steps (for details please see Fig. 5 legend).

Protocols #1 and #2 show sufficient staining of the “low expressor” positive control (>20% staining of the normal tonsillar germinal center B cells). In contrast, protocols #3 and #4 show a weak staining intensity and a reduced proportion of normal tonsillar germinal center B-cell staining. The effect on the p53 expression patterns of 5 tumors are illustrated next. The overexpression case with protocol #4 (too weak) shows a similar intensity compared with the high wild-type case stained with protocol #1 (too strong). External proficiency testing program runs have demonstrated that insufficient p53 staining was mostly due to staining being too weak (61%–84% of insufficient cases in the NordicQC run 38 and CIQC run 42) 19. Typically, these weak staining results were caused by a too low concentration of the primary antibody, including ready to use protocols not properly calibrated by the vendors (protocol #3 is a vendor protocol). The complete absence case with protocols #3 and #4 was rendered uninterpretable due to the lack of an intrinsic positive control. The cytoplasmic case is potentially misinterpreted as wild-type with protocols #3 and #4. Although the overexpression case with protocol #3 seems to stain sufficiently, protocol 3 is inferior in recognizing complete absence and cytoplasmic patterns, again highlighting the limitation on reliance of a “high expressor” sample as the sole control.

In a minority of cases, errors occur as a result of too strong staining, typically caused by an inappropriately high concentration of the primary antibody in combination with inappropriate amplification steps. This can result in a low-grade endometrioid carcinoma, and sometimes even a benign lesion, being interpreted as abnormal overexpression when it is wild-type. However, with protocols generally changing to a stronger staining for consistent detection of the internal controls, the interpretation threshold also needs to be adapted. Cut-off recommendations for overexpression versus wild-type staining are difficult to make, whereas p53 staining intensity varies across laboratories. One of the authors (M.K.) routinely utilizes a very strong protocol (#2), which is more resilient against pretreatment influence. As a consequence the staining intensity between paired endometrial biopsies and hysterectomy specimens is similar. In cases of overexpression, this results in strong staining in virtually 100% of tumor cell nuclei in a well-fixed case and at least 80% of tumor cell nuclei in a less well-fixed case. Increased cytoplasmic background in an overexpression case is generally an indication that the staining is becoming too strong, while lack of consistent detection of the internal control is an indication of too weak staining.

## PRACTICAL USES OF P53 IMMUNOHISTOCHEMISTRY

The value of p53 staining in a diagnostic sense in endometrial carcinomas is discussed in several other papers in this review and only a few brief points are made here. In endometrial carcinomas, p53 immunohistochemistry may be useful in diagnosing a high-grade carcinoma associated with unfavorable outcome and can be used as part of a panel for histotyping. However, 1 point worth mentioning is that a small percentage of low-grade endometrioid adenocarcinomas contain *TP53* mutations and exhibit mutation-type immunoreactivity. However, that being said, mutation-type p53 staining may be helpful in avoiding underdiagnosis of a serous carcinoma with intermediate-grade nuclear features as grade 1 or 2 endometrioid carcinoma 3,20. Abnormal p53 staining alone is not sufficient for the differential diagnosis of endometrioid from serous carcinomas as a significant minority of grade 3 endometrioid carcinomas show mutation-type p53 immunoreactivity 21. Grade 3 endometrioid carcinomas with mutation-type p53 expression have a worse prognosis than grade 3 endometrioid carcinomas with wild-type expression 22,23. Mutation-type p53 immunostaining serves as an indicator of the Cancer Genome Atlas (TCGA)-based molecular subtype of endometrioid carcinoma with the worst prognosis, especially when applied as part of a diagnostic algorithm 1,2.

Given that we know that the sensitivity of p53 immunohistochemistry in detection of *TP53* mutation is not 100% (see earlier), there will be a small percentage of morphologically prototypical endometrial serous carcinomas that exhibit a wild-type pattern of p53 immunoreactivity but still harbor a *TP53* mutation (eg, truncating), and the diagnosis of serous carcinoma can be made in a tumor with wild-type p53 staining. In conjunction with mismatch repair deficiency, we use p53 to classify ambiguous potentially mixed serous/endometrioid carcinomas into either category 15. Mutation-type p53 staining makes a diagnosis of dedifferentiated/undifferentiated endometrial carcinoma unlikely, although we have encountered 1 case of a *TP53* mutated grade 3 endometrioid carcinoma that underwent dedifferentiation secondary to alterations in the SWI/SNF complex 24.

## CONCLUSION

In conclusion, p53 is perhaps the single most important immunohistochemical stain used in the pathologic workup of endometrial carcinomas. Careful attention to laboratory protocols, including adequate controls, and training in interpretation is needed to achieve high interobserver consistency to make this a reliable test informing endometrial carcinoma diagnosis and subsequent management.
